# Time series analysis of new HIV diagnoses in France from 2012 to 2022

**DOI:** 10.1017/S0950268825100976

**Published:** 2026-01-07

**Authors:** David Kelly, Amber Kunkel, Lauriane Ramalli, Anna Mercier, Florence Lot, Françoise Cazein

**Affiliations:** 1 Santé publique France, Marseille, France; 2 ECDC Fellowship Programme, Field Epidemiology path (EPIET), ECDC, Stockholm, Sweden; 3 HIV, STI and Viral Hepatitis Unit, Santé publique France, Saint Maurice, France; 4 Sexual Health Unit, Santé publique France, Saint Maurice, France

**Keywords:** HIV infection, interrupted time series analysis, men who have sex with men (MSM), population surveillance, pre-exposure prophylaxis (PrEP)

## Abstract

In France, HIV prevention measures including HIV testing, treatment, and uptake of pre-exposure prophylaxis (PrEP), have increased throughout the last decade. To analyse their impact, we performed a time series analysis of monthly HIV diagnoses reported via the national HIV surveillance database. In addition, we compared the timing of HIV promotional campaigns with monthly trends in HIV testing and PrEP initiation. From January 2012 to December 2022, new HIV diagnoses steadily decreased among men who have sex with men (MSM) born in France and heterosexuals born in France, whereas HIV diagnoses increased among MSM born abroad. HIV testing activity and PrEP use in France both steadily increased from 2014 to 2020, during which multiple campaigns targeting HIV testing and prevention occurred. The decline in HIV diagnoses among MSM born in France preceded the introduction of PrEP in 2016 and continued post-2016 without any acceleration in the rate of decline. Increased awareness of, access to and uptake of HIV prevention measures remain essential to progress towards HIV elimination in France, especially among MSM born abroad.

## Background

The epidemiology of HIV in France and other European Economic Area (EEA) states has changed considerably in the last decade [1]. The increased access to testing and antiretroviral treatment (ART), prevention campaigns, and introduction of pre-exposure prophylaxis (PrEP) in 2016 in France [2], as HIV prevention measures have likely contributed to the observed HIV epidemiology in France during the last decade [1]. In France, the estimated number of new HIV diagnoses decreased moderately from 6,396 in 2012 to 5,750 in 2022 [3]. Nevertheless, the trend has not been uniform nor consistent among four main subpopulations in France: men-who-have-sex-with-men (MSM) born in France, MSM born abroad, heterosexuals born in France, and heterosexuals born abroad.

The temporal evolution of HIV incidence is complex to interpret when analysing annual HIV diagnoses in the general population. Overall annual HIV diagnoses may mask the impact of public health prevention measures on new HIV infections among different subpopulations and may be distorted by reporting delays. A time series analysis of the trends in new HIV diagnoses among the main subpopulations diagnosed with HIV, may allow us to assess the potential impact of prevention measures. The median delay estimated between HIV infection and diagnosis is 1.9 years in France (range 0.6–4.8 years), published in the National Surveillance report for 2023 [4]. Our time series analysis is over an 11-year period, which we deem sufficient to allow for the lag period associated with diagnostic delay of new HIV infection.

We aimed to measure the impact of public health prevention measures (testing activity, PrEP use, and targeted HIV prevention campaigns) on the temporal trend in new HIV diagnoses among the main subpopulations outlined above in France from 2012 to 2022 in order to better inform health policy for promotion and prevention of HIV.

## Methods

We first performed a time series analysis of the monthly number of HIV diagnoses from January 2012 to December 2022 among four main subpopulations classified by mode of transmission and country of birth: MSM born in France, MSM born abroad, heterosexuals born in France, and heterosexuals born abroad. We then assessed the impact of HIV prevention measures including testing activity, PrEP use, and targeted prevention campaigns, on these trends.

### Data sources

We analysed data from the French national electronic notifiable disease database (eDO) for HIV diagnoses among four main subpopulations from January 2012 to December 2022. To account for incomplete reporting of HIV diagnoses to the national notification system, the reported monthly number of HIV diagnoses are systematically adjusted according to the external completeness of the notification system, calculated by comparing the eDO with laboratory and hospital HIV testing data in France [3]. The internal completeness of the individual declarations reported to the notification system is then corrected by multiple imputation. To do so, a two-stage multiple imputation by chained equations procedure is applied to 29 variables (e.g., clinical stage, country of birth, probable mode of transmission, presence and, when applicable, dates of previous positive and negative HIV tests) that are missing from some notifications. Fifteen imputed databases are generated in the first phase and five in the second for a total of 75 imputed bases, and the results are combined according to Rubin’s rules [3].

We analysed HIV testing data from the French National Health Data System (SNDS) from January 2014 to December 2022, which includes the monthly number of HIV tests performed and reimbursed in the general population, excluding testing performed in the hospital inpatient and voluntary or ad hoc community settings [5]. PrEP use data were analysed using the Epi-Phare national database containing the monthly and cumulative number of PrEP initiators in France from January 2016 to December 2022 [6]. Santé publique France provided information relating to the number of HIV prevention campaigns, by month of launch and key population.

### Statistical analysis

We performed a time series analysis of monthly HIV diagnoses for each main subpopulation using a linear regression model and locally estimated scatterplot smoothing (LOESS) technique to assess the trends from 2012 to 2022 [7, 8].

We superimposed the chronology and the key population of HIV prevention campaigns against the monthly number of HIV tests (general population) from 2014 to 2022 using LOESS smoothing, and the number of PrEP initiators from 2016 to 2022 in order to assess their impact.

We plotted the monthly cumulative number of PrEP initiators with the monthly number of HIV diagnoses among MSM born in France (main subpopulation eligible) to assess overall temporal trends.

We performed an interrupted time series using a spline model to analyse the impact of monthly number of PrEP initiators on the number of HIV diagnoses among MSM born in France. We restricted the analysis to the period January 2012–December 2022, based on the observed temporal trends in HIV diagnoses among MSM born in France. The spline term was a linearized input covariable for PrEP coverage beginning month of January 2016, and a counterfactual scenario based on the pre-2016 trend in HIV diagnoses was produced.

We repeated the interrupted time series model by restricting the analysis to HIV diagnoses among MSM born in France diagnosed with symptomatic primary HIV infection as assessed by the declaring physician (henceforth ‘acute infection’), in order to the assess the potential impact of PrEP on new HIV infections, and to account for the effect of delayed HIV diagnosis in reported surveillance data. All statistical analyses were performed using R studio version 4.3.1.

## Results

From 2012 to 2022, an estimated total of 76, 396 new HIV diagnoses were reported in France. These numbered 30,183 (40%) among heterosexuals born abroad; 25,836 (34%) among MSM born in France; 12,813 (17%) among heterosexuals born in France; and 7,564 (10%) among MSM born abroad. In 2022, the estimated total number of new HIV diagnoses was 5,738 (95% CI 5,588–5,888). Of these, 38% (CI 37–40%) were heterosexuals born abroad, 27% (CI 25–29%) were MSM born in France, 16% (CI 14–17%) were heterosexuals born in France, and 13% (CI 12–15%) were MSM born abroad [3].

### Temporal trends in HIV diagnoses in France among main subpopulations

**Figure 1.**
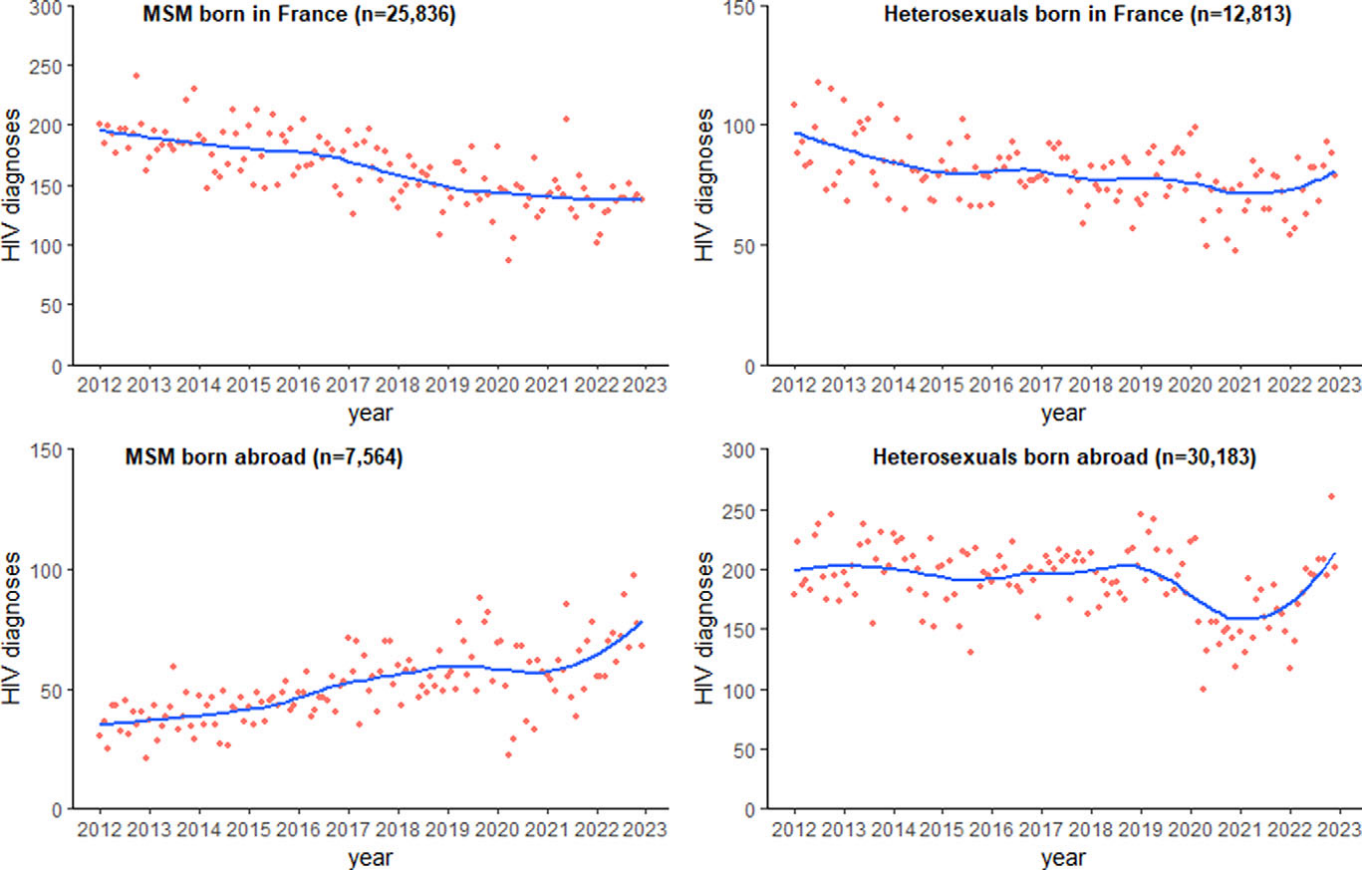
Time trends in monthly number of new HIV diagnoses in France among four main subpopulations based on mode of transmission and country of birth from January 2012 to December 2022 (n = 76,396).

The linear regression models demonstrated a significant decline in monthly HIV diagnoses in MSM born in France (coefficient = −0.013, adjusted R^2^ = 0.42) and in heterosexuals born in France (coefficient = −0.005, adjusted R^2^ = 0.29) over the study period (Table 1). This is equivalent to an average decrease of 4.7 HIV diagnoses among MSM born in France, and 1.8 HIV diagnoses among heterosexuals born in France, every year over the 11-year period. HIV diagnoses among MSM born in France showed a marked decline from 2016 to 2019 before entering a plateau phase from 2020 to 2022 (Figure 1). A more modest decline was observed among heterosexuals born in France, before entering a plateau phase from 2015 to 2021. A significantly increasing trend was observed for monthly number of HIV diagnoses in MSM born abroad (coefficient = 0.008, adjusted R^2^ = 0.42), equivalent to an average increase of 2.9 HIV diagnoses every year, with a similar plateau phase observed in 2020–2021, reaching a peak in 2022. There was a statistically significant decline in monthly HIV diagnoses in heterosexuals born abroad, observed over the study period. However, the model does not explain the data variability well (R^2^ = 0.17) and this may not be significant finding from a public health perspective, apart from a marked decrease in diagnoses during 2020 and 2021.

**Table 1.** Linear regression model estimates for the monthly number of HIV diagnoses among four main subpopulations in France from January 2012 to December 2022

Model	Intercept	Coefficient	Lower 95% CI	Upper 95% CI	Standard error	*p*-value	Adjusted R^2^
MSM born in France	379.1	−0.013	−0.015	−0.010	0.001	< 0.001	0.418
MSM born abroad	−86.6	0.008	0.007	0.009	0.001	< 0.001	0.512
Heterosexual born in France	175.4	−0.005	−0.007	−0.004	0.001	< 0.001	0.294
Heterosexual born abroad	345.2	−0.009	−0.012	−0.006	0.002	< 0.001	0.168

### Impact of HIV promotional campaigns on uptake of HIV prevention measures

**Figure 2.**
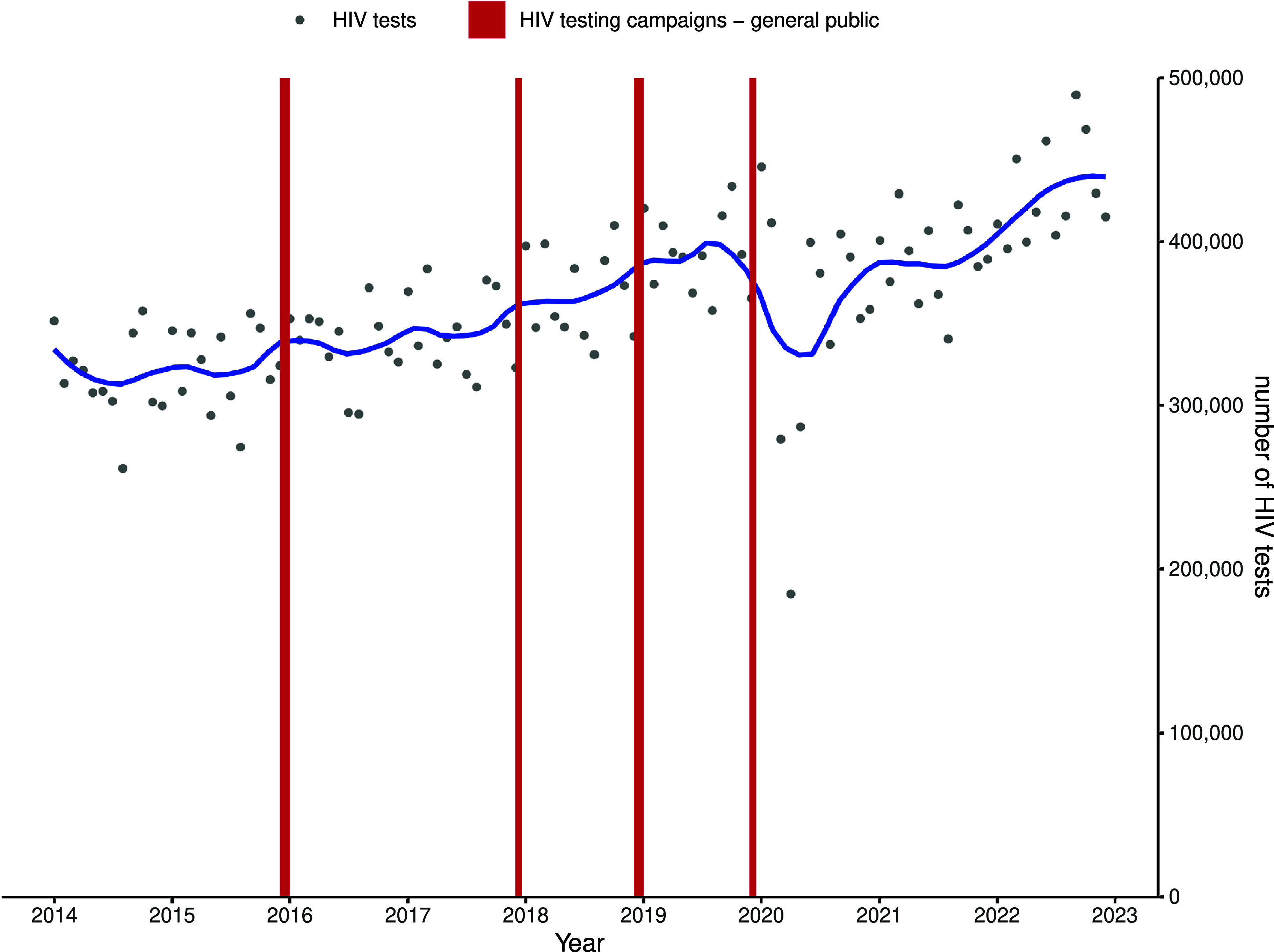
Trend in monthly number of all reimbursed HIV tests following HIV testing promotional campaigns aimed at the public in France, January 2014 to December 2022.

Overall, HIV testing activity progressively increased from 2014, reaching a peak of 490,000 monthly tests in September 2022. Minor seasonal variations were observed, with transiently higher number of tests performed in winter months. The decrease in testing activity observed in 2020 recovered to pre-2019 levels in 2021 (Figure 2).

**Figure 3.**
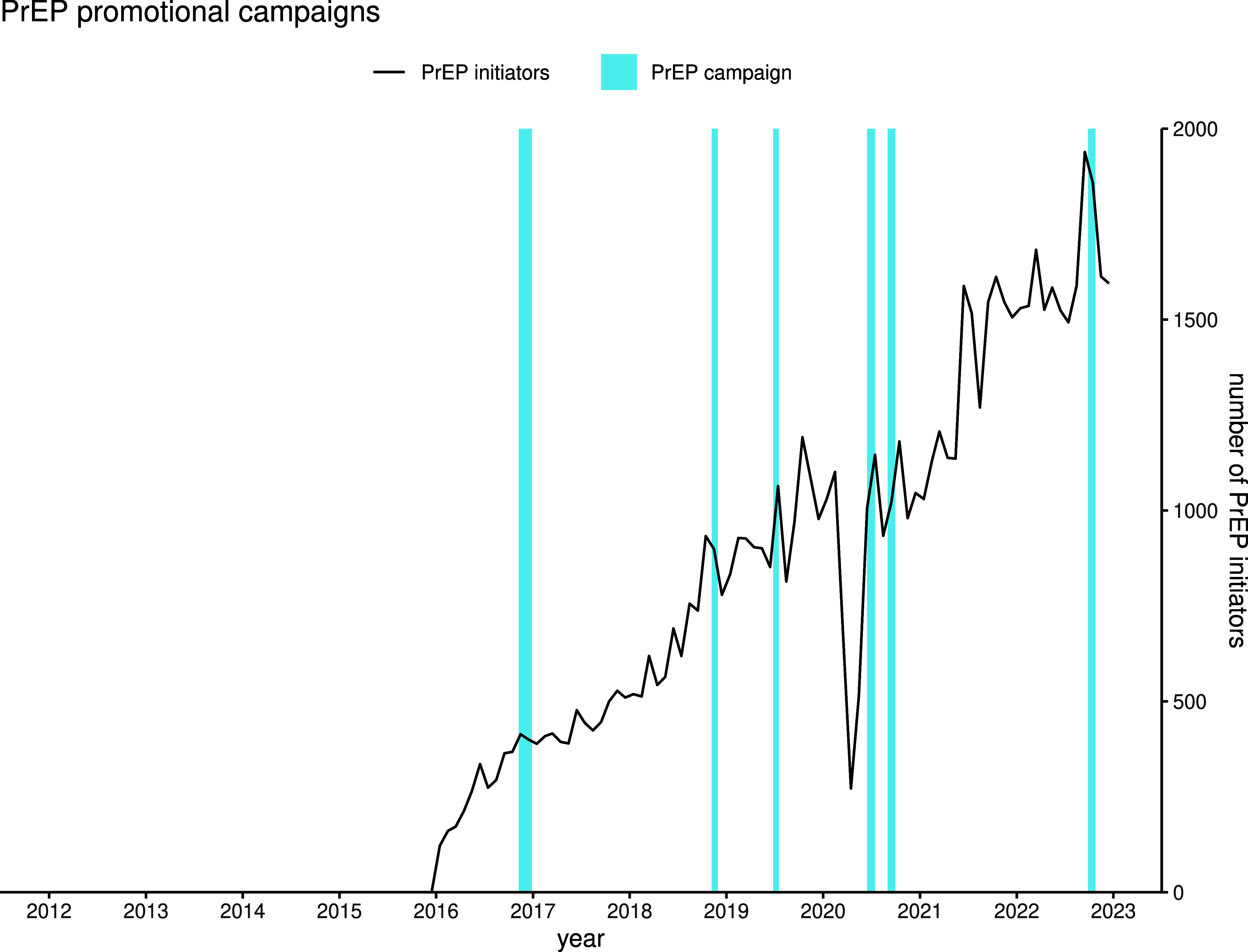
Trend in monthly PrEP initiators following the chronology of PrEP promotional campaigns in France, January 2016 to December 2022.

A steady increase in the monthly number of PrEP initiators was observed from 2016, reaching a peak of 1,900 monthly initiators in September 2022 (Figure 3). The cumulative number of PrEP initiators in France totalled 79,385 by December 2022. The trend was interrupted in 2020, with a sharp decline in the number of monthly initiators, before increasing substantially in 2021 and reverting to 2019 levels. Substantial month-to-month variability is also apparent, and PrEP promotional campaigns appear to frequently have coincided with brief spikes in monthly initiators.

### *Trend in monthly number of HIV diagnoses* versus *monthly PrEP initiators in France*

**Figure 4.**
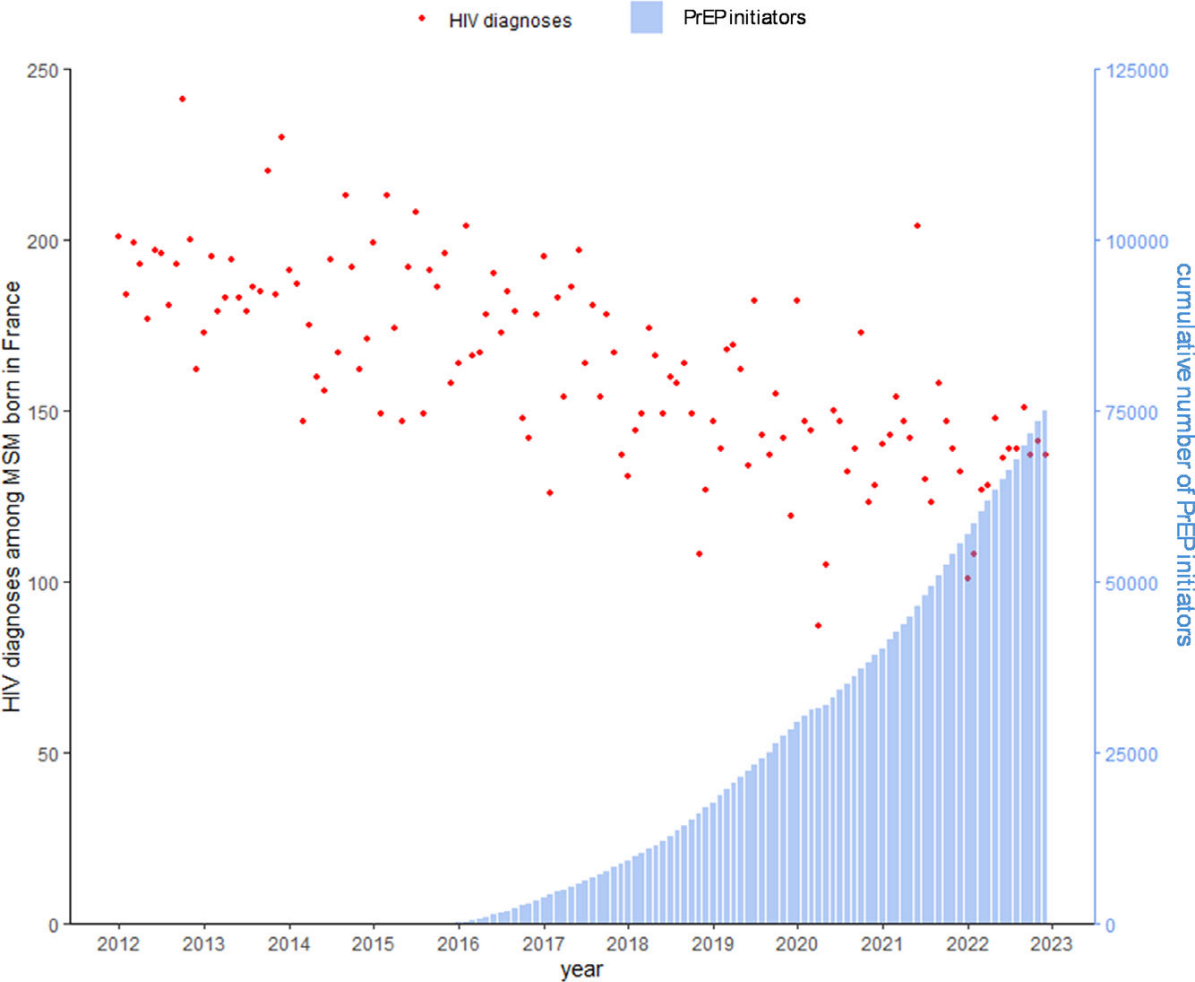
Monthly number of HIV diagnoses among MSM born in France and cumulative number of PrEP initiators in France, January 2012 to December 2022.

We observed an overall decline in number of monthly HIV diagnoses among MSM born in France from 2012 to 2022, while the cumulative number of PrEP initiators in France increased steadily from 2016, reaching 75,000 in December 2022. The decline in HIV diagnoses among MSM born in France preceded the introduction of PrEP in 2016 and continued to decline without any obvious additional decrease in HIV diagnoses during the period of increasing PrEP initiators (Figure 4).

### Impact of PrEP on HIV diagnoses among MSM born in France: Counterfactual scenario

**Figure 5.**
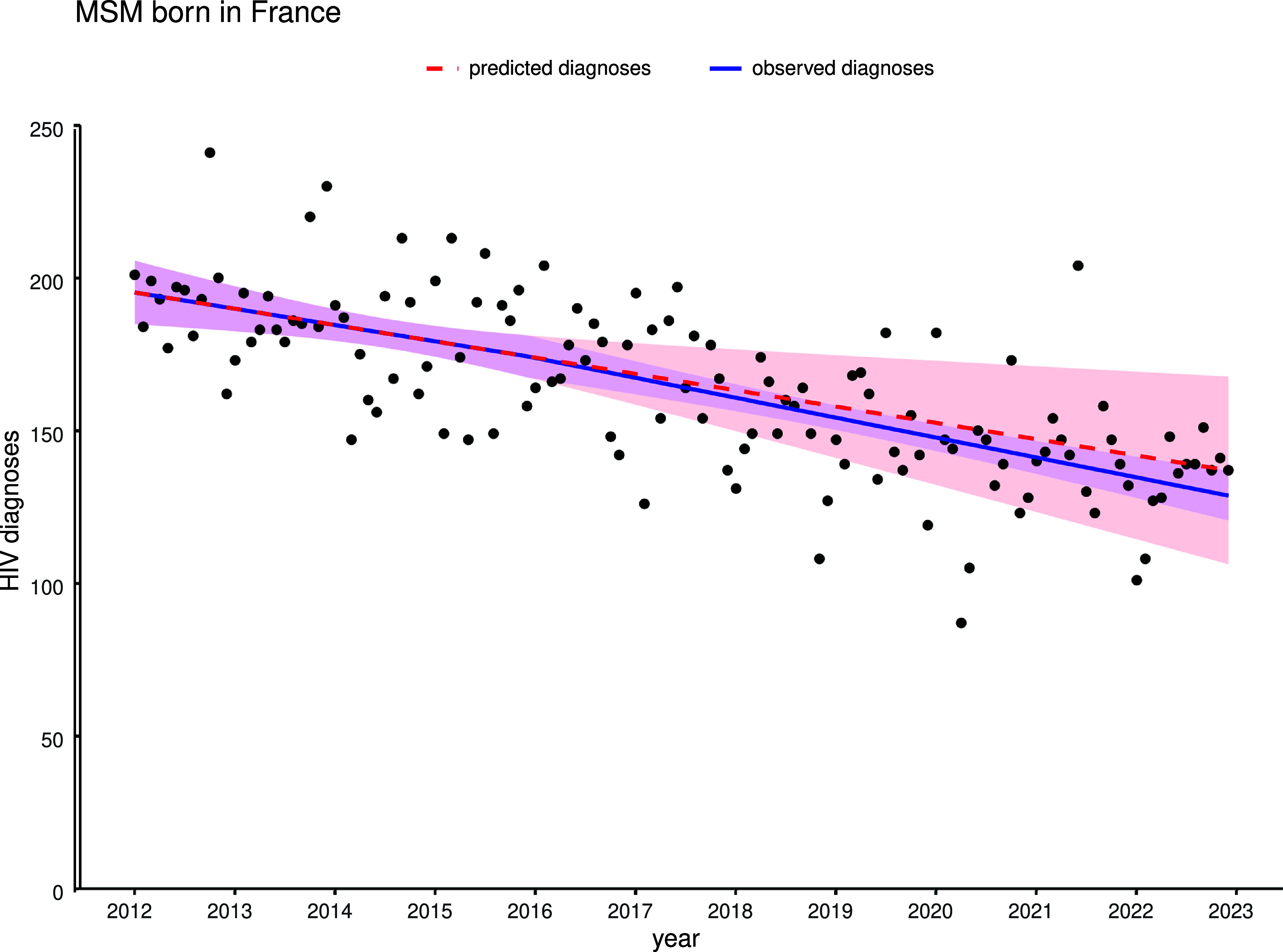
Interrupted time series analysis of observed versus expected monthly number of HIV diagnoses among MSM born in France, from January 2012 to December 2022, with counterfactual model based on pre-2016 trend.

Comparing the modelled 2012–2016 trend before the intervention of PrEP with the spline model trend demonstrated a non-significant lower number of observed versus expected HIV diagnoses from 2016 to 2022 (coefficient − 0.098, *p* = 0.64, adjusted R^2^ = 0.49). HIV diagnoses thus continued to decline, but at no greater rate than before January 2016 following PrEP introduction in France (Figure 5). Subtracting the observed from the predicted number of monthly HIV diagnoses in the counterfactual scenario, the estimated number of HIV cases potentially avoided which may be attributed exclusively to the intervention of PrEP in France was non-significant (n = 351 (95% CI [−1,001–1999]). A sub-analysis of diagnoses of acute HIV infections in MSM born in France using the same spline model demonstrated no significant impact of PrEP (coefficient = −0.038, *p* = 0.65, R^2^ = 0.15) on the decreasing trend post-2016 (Supplementary Figure S1).

## Discussion

### Epidemiological trends in HIV diagnoses in France

New HIV diagnoses have decreased overall among MSM, and heterosexuals born in France, while the trend for MSM born abroad has increased since 2012. The temporal trends in HIV diagnoses among MSM born in France showed an accelerated decline from 2013 to 2019, before entering a plateau phase. The relatively constant trend observed among MSM born abroad exhibited a similar plateau phase during 2019 and 2020. The trend among the heterosexual subpopulation born abroad showed considerable variation. These diverging trends among the four different subpopulations reflect different HIV epidemics, transmission networks, migration patterns, and access and uptake of prevention and treatment of HIV in France.

The COVID-19 pandemic likely impacted the number of HIV diagnoses reported in 2020 and 2021, due to the disruption of testing services and reporting. This effect can be observed for several months in 2020, where a considerably lower number of HIV diagnoses were reported. Nevertheless, the effect of the pandemic seems confined to 2020, with the post-2021 trends among MSM born in France, MSM born abroad and heterosexuals born in France appearing quickly recovered their pre-2020 trend, suggesting only a transient effect on HIV testing services during the pandemic period. This is potentially explained by the chronic disease epidemiology of HIV infection, whereby some HIV infections undiagnosed in 2020 due to the pandemic constraints were likely subsequently diagnosed and reported in 2021 and 2022. Additionally, border closures and travel restrictions in 2020–2021 reduced immigration, and likely resulted in a decrease in the number of people diagnosed with HIV in France who were born abroad. This likely explains the sharp decrease in HIV diagnoses in heterosexuals born abroad, and the plateau phase observed in the rising trend among MSM born abroad, during the 2020–2021 period.

Similar trends in HIV diagnoses to those observed in France have been reported by other EEA states including Germany, Netherlands, Ireland, and Spain since 2010 [9–12]. An ECDC study of all 30 EEA countries reported a progressive decline in HIV diagnoses among both MSM and heterosexual subpopulations for the period 2013–2019, with the decline beginning later among MSM in 2016 [13].

### Impact of HIV prevention measures (promotional campaigns, testing activity, PrEP) on HIV diagnoses

The immediate impact of occasional promotional campaigns on uptake of HIV prevention measures, such as testing activity and PrEP initiation in France is difficult to interpret from a visual inspection of monthly trends. Repeated promotional campaigns may have contributed to the progressive increase in uptake. This is evidenced by the sustained increase in both testing activity and PrEP initiators during the study period, following regular HIV testing and PrEP promotional campaigns in France. More detailed statistical and counterfactual analyses are needed to assess the real impact of these campaigns.

MSM and heterosexuals born in France are more likely to be aware of and have access to HIV prevention measures, such as HIV testing and ART, owing to an educational and socio-economic advantage for accessing the healthcare system compared to those born abroad in low- and middle-income countries. Among heterosexuals born abroad diagnosed with HIV in 2022, 74% were born in sub-Saharan Africa and 26% in other regions. Among MSM born abroad and diagnosed with HIV in 2022, 34% were born in sub-Saharan Africa, 19% in the Americas, 17% in Europe, and 30% in other regions [3]. Thus, the population born in France may be less likely to be exposed to HIV compared to those born abroad, and more likely to be diagnosed and treated if infected, with resulting effect on HIV transmission in these subpopulations [14]. The sustained decline in HIV diagnoses in the population born in France since 2013 may also have been aided by the progressive increase in HIV testing activity in France from 2014 to 2020, and thus treatment of people diagnosed and living with HIV.

A note of caution remains for the heterosexual population born in France, where the decreasing trend in HIV diagnoses has since entered a plateau phase from 2016 to 2022. This may reflect declining perception of HIV risk, changes in sexual prevention practices such as condom use, or increased diversity and frequency of sexual partners. This subpopulation, excepting sex workers and people who inject drugs considered on a case-by-case basis, has largely remained ineligible for PrEP in France owing to lower risk of HIV, and thus may not have benefitted from its effect on HIV transmission at a population level, compared to the MSM subpopulation [15, 16].

The trend of rising HIV diagnoses in MSM born abroad may reflect increased migration of MSM born abroad to France. Nevertheless, a recent study demonstrated a high proportion of HIV acquisition in France among in migrant MSM, indicating a true increase in HIV epidemiology rather than being due to an increase in the MSM population born aboard now residing in France [17]. Migrants are equally less likely to be familiar with accessing the healthcare system in France, may face cultural, linguistic and administrative barriers, and may suffer from HIV stigma if migrating from a country facing persecution of sexual minorities. A study of PrEP prescribers in France identified lack of awareness and interest in PrEP and right of access to free healthcare, as the main barriers to PrEP uptake [15]. These factors may reduce the likelihood of MSM born abroad accessing and engaging with HIV prevention measures such as testing, treatment and PrEP, and thus increase their risk of acquiring HIV. These underlying sociodemographic differences may explain the diverging trends in HIV diagnoses in France between MSM born in France and MSM born abroad.

### Potential impact of PrEP on HIV diagnoses in MSM born in France

The observed decline in number of HIV diagnoses among MSM born in France shows a progressive trend between 2013 and 2020. The start of the decline in 2013 may have corresponded to a change in clinical practice following the update to HIV guidelines recommending early initiation of ART at diagnosis, rather than at a specific CD4 count cut-off [18]. The resulting increase in viral suppression at a population level may have thus contributed to reducing the risk of secondary transmission from MSM born in France diagnosed with HIV from 2013 onwards.

Additionally, increased testing activity observed in France since 2014 may have enabled more MSM to be diagnosed and initiate treatment, thereby reducing transmission from MSM with previously undiagnosed HIV. MSM constitute the key population eligible for PrEP since its rollout in France in January 2016 [16], with men accounting for 97% of PrEP users according to SNDS reimbursement data from 2016 to 2023 [2, 19]. The proportion of MSM among migrant users of PrEP declined in France from 65% in 2018 to 38% in 2021 [15], suggesting that MSM born in France remain the primary population accessing and utilising this prevention measure [20].

Based on the counterfactual model in our study, the introduction of PrEP in 2016 likely contributed to sustaining the pre-existing decline since 2013 in HIV diagnoses observed among the MSM subpopulation born in France beyond 2016. PrEP alone does not appear to have a significant additional impact on the rate of decline in HIV diagnoses among MSM born in France since 2016 and likely represents a modest effect at population compared to individual level. Our counterfactual model did not account for other factors influencing HIV transmission such as testing or treatment coverage, or changing sexual risk behaviours such as condom use or serosorting, which may have had an impact at population level.

Our study reports an increasing monthly number of PrEP initiators but lacks an estimation of PrEP coverage in the eligible MSM subpopulation living in France. Thus, insufficient population coverage of PrEP may explain why the observed decline in HIV diagnoses at population level has been more modest than predicted by efficacy in clinical trials. The proportion of sexually active MSM reporting PrEP use at last sexual encounter (28%) appears to represent as yet insufficient uptake of this prevention measure among the key population in France to achieve HIV elimination [20, 21]. Despite ECDC data for the period 2019–2022 reporting the number of PrEP users in France (47071) exceeded that in Germany (30000) and in Spain (18000), the elevated number of users likely represents insufficient PrEP coverage among MSM to have the same real-world effectiveness against HIV infection at population level in France compared to the clinical efficacy demonstrated in clinical trials [22, 23]. A further potential explanation for the modest decrease is that those MSM at highest risk of HIV infection may not constitute the primary user population of PrEP. Additionally, suboptimal adherence to PrEP dosing may also limit the effectiveness in preventing HIV transmission [22]. Reinforcing health promotion efforts to increase PrEP initiation and adherence among MSM in France would likely result in a further reduction in HIV incidence and decline in HIV diagnoses in this subpopulation [24].

A further potential explanation for the observed modest impact of PrEP on HIV diagnoses is the changing methods of protection among sexually active MSM in France reported by the ERAS survey [25]. A decline in systematic condom use was reported from 46% in 2017 to 28% in the 2023 ERAS survey, likely corresponding to increased use of PrEP as an effective HIV prevention method among MSM [26]. Condomless anal intercourse (CAI) was increasingly reported by sexually active MSM from 39% in 2017 to 58% in the 2023 ERAS survey [26]. In the absence of concomitant PrEP use, CAI confers a higher risk of HIV infection than consistent condom use. Furthermore, the corresponding reported increased use of PrEP from 5% in 2017 to 25% in 2023 may not necessarily occur among the same individual MSM who report reduced condom use, with implications for risk of HIV infection. The increase in CAI may thus have counterbalanced the protective effect of increased PrEP users in the MSM *s*ubpopulation since 2016, if the reported CAI was unaccompanied by PrEP use at the individual level. The observed lack of additional effect of PrEP on acute HIV infections diagnosed is unexpected; however, our sub-analysis is based on smaller monthly numbers.

The population level impact of PrEP on HIV diagnoses among MSM born in France from our study mirrors the trends observed elsewhere in Europe. National HIV surveillance reports in the Netherlands and Ireland covering the period up to 2022, and a surveillance study in Spain for the period 2014–2019 all reported a decline in HIV diagnoses among MSM, which preceded the introduction of PrEP by national health services in 2016 in the Netherlands, and in 2019 in Ireland and Spain [10–12]. A study limited to MSM attending sexual health clinics in Scotland for the period 2015–2019 reported a significant decrease in HIV incidence in in the period after 2017 corresponding to PrEP availability [27]. The joint ECDC WHO EURO report on HIV/AIDS surveillance from 2013 to 2022 demonstrated a progressive decline in HIV diagnoses among MSM in the EEA. The decline began in 2014, without any additional effect on the rate of decline observed for the period corresponding to PrEP availability in many EEA countries [1].

### Strengths and limitations

Using 11 years of data from the French national HIV database has permitted us to perform a robust time series analysis to identify diverging trends in HIV epidemiology among four main subpopulations, and to assess the impact of HIV prevention measures at population level in France, using the best available national data sources on HIV testing, HIV diagnoses, and PrEP use.

The surveillance data used for our time series analysis consists of reported HIV diagnoses, rather than HIV infections in France. The median delay between HIV infection and diagnosis is estimated to be 2.2 years, by a Netherlands study [28]. Therefore, the impact of PrEP on new HIV infections in France may not be observed among the number of reported HIV diagnoses reported via the surveillance system in France until 2–3 years later. This time lag is an inherent limitation of analysing HIV diagnoses, owing to inability to estimate true HIV infections in a given year. Nevertheless, our sub-analysis limited to diagnoses of acute HIV infections indicated no demonstrable change following PrEP introduction. Equally, our time series analysis of aggregate population data did not account for whether individuals diagnosed with HIV were aware of, had access to or were users of prevention measures such as PrEP.

The COVID-19 pandemic resulted in reduced HIV testing activity and reduced migrant arrivals in France in 2020, which disrupted the trends in HIV diagnoses reported during this period among the four main subpopulations. Equally, a ‘catch-up effect’ on HIV diagnoses may explain the rise observed in 2022 among heterosexuals born in France and MSM born abroad, following a reduction in testing activity in 2020. This effect remains to be confirmed by HIV diagnoses data from 2023.

The external completeness of reporting HIV cases to the national notification system is a limitation of the analysis, estimated for recent years at 54% in 2020, 57% in 2021, and 57% in 2022 [3]. External completeness is adjusted for by the methods described previously [3], it is, however, estimated annually and not monthly, which may lead to under- or over-reporting of monthly HIV diagnoses. Additionally, low internal data completeness may result in an overestimation or underestimation of HIV diagnoses according to the mode of transmission or country of birth; an inherent limitation of using multiple imputation as a correction method for missing data. Nevertheless, our time series analysis is based on 11 years of national data, somewhat reducing thus these limits.

Other factors related to HIV testing and thus reported HIV diagnoses may equally have contributed to the observed trends, including increased community-based testing, outreach clinics, and self-testing kits in France since 2010 not captured by SNDS data Our counterfactual model for the impact of PrEP did not account for these additional factors.

## Conclusion

This time series analysis of HIV diagnoses using national surveillance data illustrates the complex and divergent trends in HIV epidemiology among different subpopulations in France since 2012. The findings notably demonstrate a sustained decline in HIV diagnoses among both MSM and heterosexuals born in France for the period of 2012–2022. PrEP introduction likely contributed to sustaining the pre-existing steady decline in HIV diagnoses observed among the subpopulation of MSM born in France, without accelerating the rate of decline. Applying similar methods to HIV incidence estimates could help to confirm these results. Increasing awareness, acceptance, and uptake of HIV prevention measures are essential to reducing the number of HIV diagnoses, especially with respect to MSM born abroad, in whom HIV diagnoses are increasing, in order to progress HIV elimination in France.

## Supporting information

10.1017/S0950268825100976.sm001 Kelly et al. supplementary material Kelly et al. supplementary material

## Data Availability

Data are available from the authors upon request and in compliance with French data protection laws.

## Supplementary material

The supplementary material for this article can be found at http://doi.org/10.1017/S0950268825100976.

## References

[1] European Centre for Disease Prevention and Control (2023) WHO Europe Region,. HIV/AIDS surveillance in Europe. https://www.ecdc.europa.eu/en/publications-data/hivaids-surveillance-europe-2023-2022-data.

[2] Billioti de Gage S, Desplas D and Dray-Spira R (2022) Roll-out of HIV pre-exposure prophylaxis use in France: A nationwide observational study from 2016 to 2021. Lancet Regional Health Europe. 22, 100486. 10.1016/j.lanepe.2022.100486. 35990255 PMC9386455

[3] Santé publique France (2023) Bulletin de santé publique VIH-IST. Novembre 2023. Annexe 2. https://www.santepubliquefrance.fr/maladies-et-traumatismes/infections-sexuellement-transmissibles/vih-sida/documents/bulletin-national/bulletin-de-sante-publique-vih-ist.-novembre-2023.

[4] Santé publique France (2024) VIH et IST bactériennes en France. Bilan 2023. https://www.santepubliquefrance.fr/maladies-et-traumatismes/infections-sexuellement-transmissibles/vih-sida/documents/bulletin-national/vih-et-ist-bacteriennes-en-france.-bilan-2023#:~:text=Le%20nombre%20de%20personnes%20ayant,forte%20baisse%20observ%C3%A9e%20en%202020.

[5] Kounta CH, et al. (2022) HIV and bacterial sexually transmitted infections screening in France, 2014-2021. Bulletin Epidémiologique Hebdomadaire. 2022;(24-25), 456–62. http://beh.santepubliquefrance.fr/beh/2022/24-25/2022_24-25_4.html

[6] Epi-Phare Edpds (2023) Suivi de l’utilisation de la prophylaxie pré-exposition (PrEP) au VIH 2023. https://www.epi-phare.fr/rapports-detudes-et-publications/prep-vih-2023/.

[7] Jacoby WG (2000) Loess: A nonparametric, graphical tool for depicting relationships between variables. Electoral Studies 19(4), 577–613. 10.1016/S0261-3794(99)00028-1.

[8] Wickham H ggplot2: Smoothed conditional means 2016 [cited 2024]. https://ggplot2.tidyverse.org/reference/geom_smooth.html.

[9] Robert Koch Institut Germany (2023) Epidemiologisches Bulletin 47/2023. https://www.rki.de/DE/Content/Infekt/EpidBull/Archiv/2023/Ausgaben/47_23.pdf?__blob=publicationFile.

[10] Stichting HIV Monitoring (2023) Human Immunodeficiency Virus (HIV) Infection in the Netherlands. Monitoring Report 2023. https://www.hiv-monitoring.nl/application/files/1417/0229/8520/SHM_HIV_MONITORING_REPORT_NL_2023.pdf.

[11] Health Protection Surveillance Centre Ireland (2022) HIV in Ireland: Latest trends to end 2022. https://www.hpsc.ie/a-z/hivandaids/hivdataandreports/HIV_trends_to%20end%202022_final.pdf.

[12] Ayerdi Aguirrebengoa O, et al. (2021) Changes in the profile of newly HIV-diagnosed men who have sex with men, Madrid, 2014 to 2019. Euro Surveillance 26(47). 10.2807/1560-7917.ES.2021.26.47.2001501. PMC8619870 34823642

[13] Reyes-Urueña J, et al. (2023) HIV diagnoses among people born in Ukraine reported by EU/EEA countries in 2022: Impact on regional HIV trends and implications for healthcare planning. Euro Surveillance 28(48). 10.2807/1560-7917.ES.2023.28.48.2300642. PMC10690861 38037726

[14] Duracinsky M, et al. (2022) Etude de la prévalence de l’infection à VIH et des hépatites virales B et C, et chez les migrants réguliers en France: Données de l’étude STRADA (2017-2020). Médecine et Maladies Infectieuse Formation. 7622(1002), S1–S166.

[15] Cordel H, et al. (2022) La PrEP chez les migrants : y sommes-nous vraiment ? Bulletin Epidémiologique Hebdomadaire 2022;(24-25), 438–45. http://beh.santepubliquefrance.fr/beh/2022/24-25/2022_24-25_2.html.

[16] Conseil national du sida et des hépatites virales (2021) Avis sur la place de la PrEP dans la prévention du VIH en France: changer de paradigme, changer d’échelle online: CNS. https://cns.sante.fr/wp-content/uploads/2021/05/2021-04-15_avis_fr_prevention.pdf.

[17] Palich R, et al. (2024) High proportion of post-migration HIV acquisition in migrant men who have sex with men receiving HIV care in the Paris region, and associations with social disadvantage and sexual behaviours: Results of the ANRS-MIE GANYMEDE study, France, 2021 to 2022. Euro Surveillance 29(11). 10.2807/1560-7917.ES.2024.29.11.2300445. PMC10941311 38487889

[18] Morlat P, et al. Ministère des affaires sociales et de la Santé, Conseil national du Sida, Agence nationale de recherches Sur le sida et les hépatites virales. Prise en charge médicale des personnes vivant avec le VIH Recommandations du groupe d’experts Rapport 2013. https://sante.gouv.fr/IMG/pdf/Rapport_Morlat_2013_Mise_en_ligne.pdf

[19] Epi-Phare Epidémiologie de produits de santé. Suivi de l’utilisation de la prophylaxie pré-exposition (PrEP) au VIH 2023. https://www.epi-phare.fr/rapports-detudes-et-publications/prep-vih-2023/.

[20] Velter A, et al. (2023) HIV Pre-exposure prophylaxis among men who have sex with men responding to the Rapport au Sexe 2023 survey: Who is eligible? Who are the users? Bulletin Epidémiologique Hebdomadaire 2023(24–25), 542–552.

[21] Jijón S, et al. (2021) Can HIV epidemics among MSM be eliminated through participation in preexposure prophylaxis rollouts? AIDS 35(14), 2347–2354. 10.1097/QAD.0000000000003012. 34224442

[22] Jourdain H, et al. (2022) Real-world effectiveness of pre-exposure prophylaxis in men at high risk of HIV infection in France: A nested case-control study. The Lancet Public Health 7(6), e529. 10.1016/S2468-2667(22)00106-2. 35660214

[23] European Centre for Disease Prevention and Control (2024) Pre-exposure prophylaxis for HIV prevention in Europe and Central Asia Monitoring implementation of the Dublin Declaration – 2023 progress report.

[24] Billioti de Gage S, et al. (2023) Roll-out and effectiveness of HIV pre-exposure prophylaxis in France: An overview. Thérapie 78(5), 585–591. 10.1016/j.therap.2023.02.010. 36894453

[25] Velter A, et al. (2022) Trends in HIV protection methods among HIV-negative men who have sex with men: Results from the Rapport au sexe survey 2017-2019-2021, France. Bulletin Epidémiologique Hebdomadaire (24–25), 430–438.

[26] Velter A. (2024) Evolution de la couverture préventive contre le VIH en France parmi les hommes ayant des relations sexuelles avec les hommes: Enquête rapport au sexe 2017-2019-2021-2023. *Journées thématiques IST, PrEP, santé sexuelle 2024*; Paris2024.

[27] Estcourt C, et al. (2021) Population-level effectiveness of a national HIV preexposure prophylaxis programme in MSM. AIDS (London, England) 35(4), 665–673. 10.1097/QAD.0000000000002790. 33290298 PMC7924973

[28] HIV Transmission Elimination AMsterdam (H-TEAM) Initiative (2023) A 95% decline in estimated newly acquired HIV infections, Amsterdam, 2010 to 2022. Euro Surveillance 28(40). 10.2807/1560-7917.ES.2023.28.40.2300515. PMC10557385 37796442

