# Supplementary material for: Time series analysis of new HIV diagnoses in France from 2012 to 2022
Source: Epidemiol Infect. 2026 Jan 7;154:e18. doi: 10.1017/S0950268825100976 (PMC12877913; doi:10.1017/S0950268825100976)

Epidemiology and Infection

Time series analysis of new HIV diagnoses in France from 2012 to 2022

David Kelly^1, 2^, Amber Kunkel^3*^, Lauriane Ramalli^1^, Anna Mercier^4^, Florence Lot^3^, Françoise Cazein^3^

**Supplementary Material**

Figure S1: Interrupted time series analysis of observed versus expected monthly number of acute HIV infections among MSM born in France, from January 2012 to December 2022, with counterfactual model based on pre-2016 trend.


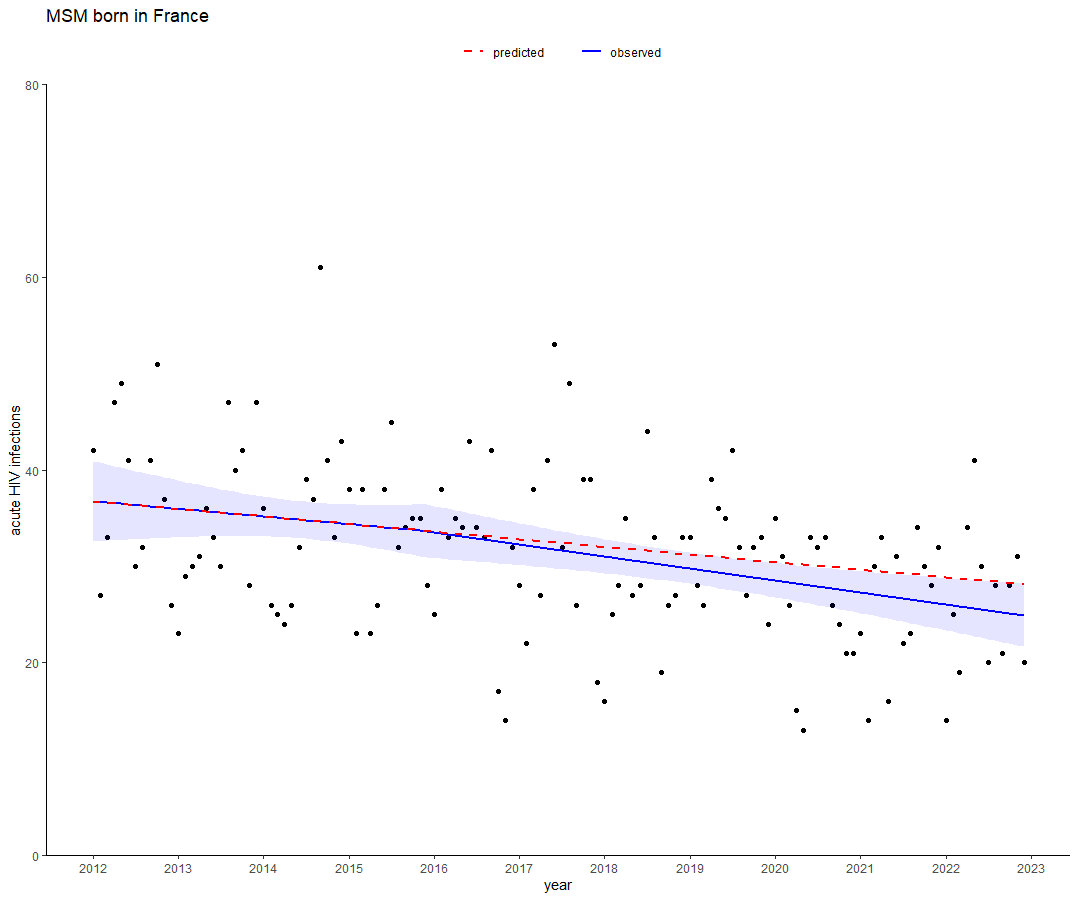

Supplement: Kelly et al. supplementary material [file S0950268825100976sup001.docx]
